# Traité des Maladies des Reins, &c.

**Published:** 1842-01-01

**Authors:** 


### Traité des Maladies des Reins, &c. Par *P. Rayer*. 3 tomes. Paris, 1839-40-41.

In our last number we submitted to our readers' attention a lengthened review of M. Rayer's Researches on that form of Renal Disease, which has of late years excited so much interest among medical men, and is known under the names of Albuminuria, Albuminous Nephritis, or Morbus *Brightii*. On the present occasion we shall complete our notice of these important volumes, by briefly analysing the chapters on the simple, the gouty, and the rheumatic forms of inflammation of the kidneys, on Pyelitis or inflammation of their pelves and calices, and on Hæmaturia, occasional and endemic.

Under the generic term of Nephritis have been comprehended all the inflammations of the various tissues which enter into the organization of the kidneys,—not only of the renal substance, properly so called, but also of its membranes, its vessels, and of the excretory ducts of the urine. It is unnecessary to say how faulty such a classification must be. As well might we groupe under one term the different kinds of pneumonia, and pleurisy and bronchitis at the same time.

M. *Rayer* has endeavoured to disentangle this confused web; and, basing his conclusions on a most elaborate examination of facts drawn partly from his own experience and partly from the writings of others, he has been led to propose the following catalogue of the inflammatory diseases of the renal organs.

1st *groupe*.—Nephritis, or inflammation of the cortical, or of the tubular substance of the kidneys.

This groupe comprises—1, simple nephritis; 2, nephritis from morbid poisons; 3, arthritic nephritis (gouty and rheumatic, &c.); 4, albuminous nephritis.

2nd *groupe*.—Pyelitis, or inflammation of the pelves and calices of the kidneys.

The principal species of this groupe are—1, simple pyelitis; 2, blenorrhagic, or gonorrheal pyelitis; 3, calculous pyelitis; and, 4, verminous pyelitis.

3d *groupe*.—Perinephritis, or inflammation of the cellular and fibrous coverings of the kidneys, or of the fatty cellular tissue which surrounds them.

The different inflammations arranged under these three heads differ from each other not only in their seat or the tissue which is mainly affected, but also in the symptoms they produce during life, and the anatomical appearances which they present upon dissection.

But as we find in certain cases of pulmonic inflammation that the parenchyma of the lungs, as well as the bronchi and the pleuræ, are all involved at the same time, so we observe that all the different tissues of the kidneys may be simultaneously affected: to such a case we should give the appellation of *Pyelo-nephritis.*

We shall begin our comments with a short notice of

*Simple Nephritis,*—as this must be considered the standard with which the other forms of renal inflammation are to be compared and contrasted. Under this term M. *Rayer* comprehends all the inflammations of the cortical and tubular portions of the kidneys, which are induced by any mechanical or accidental cause, and which are not dependent upon a constitutional disposition or diathesis or upon the action of a morbific poison.

The kidney may become inflamed in consequence of a blow or wound in the loins, or of a violent muscular effort, which has strained the lumbar muscles and powerfully contracted the abdominal parietes. The presence of a calculus or any other foreign substance; the retention of the urine in the pelvis of the organ, from an obstruction of the ureter, bladder, or urethra, or from a paralytic weakness of these organs; the action of such stimulants as cantharides, turpentine, nitre, &c.; the sudden impression of cold and moisture on the body when heated and perspiring, more especially if the patient be affected at the same time with any complaint of the excretory urinary organs;—these are the most frequent causes of simple renal inflammation. M. *Rayer* is of opinion, that nephritis from the action of cold and moisture is of much more frequent occurrence than is generally imagined. The disease is more common in the advanced than in the more youthful periods of life: this is what we might expect, considering the greater frequency of urinary complaints, as well as of cerebro-spinal affections in mature and old age.

If the diseases of the urethra, prostate gland, and bladder are frequent causes of nephritis in men, the diseases of the uterus and ovaria, the existence of abdominal tumors, not to mention gestation and delivery, are no less powerful agencies to the same effect in women. The urinary secretion in simple nephritis is usually scanty, sometimes almost completely suppressed: generally it is only slightly acid, or it may be neutral or even alkaline. In the early period of the disease it often contains a portion of blood loaded with it; and in the latter stage it occasionally contains an admixture of pus. Whenever either of these ingredients are present in the urine, this fluid may be found to exhibit signs of albumen on the application of heat or the addition of nitric acid. The temporary presence of a certain portion of albumen in the urine is by no means in itself a sign of the existence of the disease, which has been called albuminuria or albuminous nephritis; but, as we have discussed the history of this form of the disease at considerable length in the last number of this Review, it is unnecessary to revert to the subject at present. In the simple, as well as in the albuminous, form of nephritis, the proportion of the uric acid and its salts is less than in health; but the diminution is much more considerable in the latter than in the former variety of the disease.

One of the most formidable consequences of nephritis is unquestionably the great diminution, or perhaps the complete suppression, of the urinary secretions, and the cerebral symptoms which are apt to ensue upon such a condition. In certain cases of typhoid and other fevers there is a tendency to this state of things supervening. We need not add that the most prompt and vigorous treatment is required to save the life of the patient. In the worst set of cases the kidneys become more or less decidedly gangrenous: this termination of nephritis has also been observed after death from certain poisons.

The *chronic* form of simple nephritis is probably of much more frequent occurrence than the *acute*. The descriptions hitherto given of the disease by different writers have generally been far from accurate; in consequence of their confounding inflammation of the pelvis and calices with that of the tubular or cortical substance of the organ. Whenever the urine has been decidedly purulent, and pain and uneasiness in the loins, extending down along the course of the spermatic cord, have been felt, the case has been regarded as one of *nephritis;* whereas these, properly speaking, are the symptoms rather of *pyelitis* than of it.

According to the observations of M. *Rayer*, the diagnosis of chronic nephritis is often utterly impossible unless the urine is attentively examined. "Constant pains in one or in both of the renal regions, co-existing with a diminished acidity, or with a neutral, or still more with an alkaline state of the urine—whether there be a retention or not of this secretion—and with a sense of feebleness in the lower limbs, are the principal characters of the disease."*

When the urine is alkaline, it is usually opaque or cloudy, unless the proportion of the phosphatic salts is very small indeed. The sediment is generally found to be composed of the ammoniaco-magnesian phosphate, and of phosphate of lime, with or without a small quantity of the urates, and with more or less mucous matter combined. "In fine," adds M. *Rayer*, "chronic nephritis is one of the most favourable conditions for the production of phosphatic calculi."

The effects of appropriate treatment will sometimes very beautifully confirm the diagnosis which we may have formed. Thus, after the application of the cupping-glasses and the use of a mild unirritating diet, the urine, which for some time before had been thick and decidedly alkaline, may be found to become transparent and to recover its normal acidity—perhaps again to return to its unhealthy condition in consequence of some irregularity of diet, or of fatigue, or of exposure to cold, &c.

The symptoms of chronic nephritis being often so very obscure in themselves, and rendered still more so by their not unfrequent alternations of

---

* The renal pain, which by-the-bye is in most cases obscure, and is felt only when pressure is made on the loins, seldom extends, according to the experience of our author, in the direction of the ureter, and is almost never accompanied with pain in the testicles. In general, the urine is voided frequently, and only in a small quantity at a time.

amendment and aggravation, it becomes the duty of the physician to be much on his guard in his diagnosis of many cases. Often the disease has continued for months or even years, before the patient applies to a medical man for relief; and, even when he does, he is not at all aware that the primary seat of his distress is in the renal region. The pain there may be so trifling as not to attract his notice, unless firm pressure be made over it; and the increasing frequency of calls to urine may have come on so gradually that he has never thought it worth while to mention the circumstance.

When chronic nephritis has attacked both kidneys, its principal symptom is the slow and progressive deterioration of the general health. This cachectic state favours the development of many other diseases, especially obstinate catarrhs and pulmonary consumption.

M. *Rayer* describes at great length the various morbid appearances which simple nephritis, both in its acute and in its chronic form, is apt to induce in the kidneys and in their coverings; but we cannot enter into this subject, as it would require much more space than we can allow to give an accurate idea of his, perhaps too elaborate, descriptions.

After discussing the diagnosis of the disease, he makes some good remarks on the prognosis which should be formed in certain cases. He dwells particularly on the danger of nephritic attacks in patients who have long laboured under any disease of the bladder, prostate gland, or urethra; more especially if such attacks have come on after a surgical operation, such as lithotomy, lithotrity, or even the introduction of a sound. Under such circumstances the invasion of nephritis is often very sudden and proves very rapidly fatal. Many an old man, who may have been for years affected with an incontinence of urine, but whose general health had remained good, is carried off very quickly, if the kidneys once manifest the symptoms of renal inflammation. This circumstance should therefore be strongly impressed on the minds of all medical men.

M. *Rayer* alludes to the occurrence of nephritis in parturient women in the following passage :—

"In recently-delivered women, an attack of nephritis is sometimes preceded or accompanied by inflammation of the ovarian veins. The existence of pain in the renal region requires that the state of the uterus and its appendages should be examined with the greatest care. Like every other sort of inflammation occurring in parturient women, nephritis has a great tendency in them to terminate in suppuration. In spite of the fatigue and exhaustion which follow delivery, we must have recourse to free bloodletting, and this with the greatest promptitude, as the disease is often overlooked for the first day or two after its invasion; the lumbar pains having been regarded merely as one of the effects of the labour."

In all cases of chronic nephritis, from whatever cause it may proceed, there is no remedy so decidedly useful as cupping over the loins. Even when there is co-existing disease of the bladder or of some other part of the urinary apparatus, the relief is often almost instantaneous after some ounces of blood have been withdrawn by means of the scarificator. The application of hot poultices afterwards, and the use of the warm bath may generally be recommended at the same time.

With respect to the use of acids, as means to obviate an alkaline state of the urine, and to counteract the tendency to the precipitation of the phosphatic salts, M. *Rayer* informs us that his experience is by no means very favourable to the practice. He has repeatedly observed that they entirely failed in producing the desired effect, and, when continued too long, or administered in large doses, they often seemed to injure the state of the stomach and the general health of the patient.

"I have seen," says he, "in workmen affected with chronic nephritis, the urine become acid and transparent after a fortnight's repose, and enjoyment of a good diet, in conjunction with one or more applications of the cupping glasses to the loins, and again become muddy and alkaline when the food was less nourishing, or the patients had taken any extra fatigue. I have carefully compared the effects of a vegetable and of an animal diet in such cases, and am satisfied that the latter is to be preferred. Not only does the state of the urinary secretion become more normal under the use of nutritious food, but the general strength and health of the patient very decidedly improve."

When there is much distress from the frequent calls to pass water, the use of opium, of anodyne enemata, and of emollient hip baths, will often give relief for a time. The acidulated decoction of the Pareira brava, the extract of the Uva ursi combined with the extract of hop or of henbane, the infusion of carrot-seeds, or of the leaves of the Diosma crenata, have been given with benefit in some cases ; but the use of these remedies should be suspended whenever any aggravation of the inflammatory symptoms takes place.

The establishment of a purulent drain from the renal region, and the exhibition of the milder preparations of steel, have seemed to produce good effects occasionally.

M. *Rayer* has, as we have said, described at great length the various complications, which have been observed to attend occasionally the existence of nephritis. One of the most formidable of them is the development of cerebral disease, indicated by the sudden supervention of stupor and coma, which unfortunately are generally the precursors of a fatal termination. In this form of nephritis, the vomiting is more than usually severe and obstinate ; so much so as often to excite the suspicion in the mind of the medical attendant of the existence of gastritis or peritonitis. The pain, however, situated in the loins, and the diminution, or even the total suppression, of the urine, point out that the kidneys are seriously affected. Whenever there is a complete suppression of the urine—the *ischuria renalis* of authors—the tendency to cerebral disease is much to be feared.

Several cases are quoted by our author of the absence of one of the kidneys, and where the other became the seat of inflammation, with or without the presence of calculi at the same time. We cannot be surprised that under such circumstances the renal disease proves inevitably fatal. He has also collected several instances of what has been called *fusion of the* kidneys, in which the double organ became the seat of inflammation. *Vesalius* long ago remarked that lean persons, in whom the abdomen is prominent and the lumbar region depressed, are often found to have but one kidney placed transversely across the vertebral column ; and M. *Rayer* thinks that he has been able, during the life of the patients, to confirm the truth of this statement in more than one case. He adds that this peculiarity of the kidneys ought to be kept in mind by the physician, as the swelling caused by the irregularity of their position has been mistaken for a morbid tumor in the abdomen.*

*Gouty Nephritis.*

The connexion of renal inflammation with a gouty diathesis of the system has been pointed out by many practical writers from a very early period of medical literature. The well-known tendency, that exists to the deposition of sand and minute calculi not only in the pelves and calices, but sometimes even in the very substance and on the surface of the kidneys in gouty patients, is in itself sufficient to explain the frequent development of nephritis, usually of a chronic character, under such circumstances. As long as the calculi do not impede the free escape of the urine from the kidneys, the symptoms of the disease are usually indistinct and may not sufficiently attract the notice of the medical attendant; the patient complains rather of a dull heavy weight than of actual pain in the loins, extending sometimes down along the corresponding limb, and to the testicle, and attended with a sense of numbness in the same direction; the urine is almost always more decidedly acid than in health, even when its colour is not unusually deep; and the digestive functions are generally more or less disordered. The most important diagnostic symptom is unquestionably the state of the urinary secretion; and therefore, unless this be attentively examined and tested, the physician will necessarily remain in uncertainty as to the nature and cause of the malady. When first voided the urine sometimes exhibits minute grains of crystallized uric acid held in suspension in the fluid; if left for a few hours in a glass tube, we may generally detect a certain number of these crystals adhering to its inner surface. Even when the gouty diathesis is not very *prononcé*, the sediment, if examined with the microscope, often appears entirely composed of such crystals, which may, or may not, be blended with blood, or mucus or pus. This state of the urine is so inherent, so to speak, in gouty patients who suffer from renal pains, that I have known it to continue, says our author, after more than a year or two's use of alkaline baths and drinks.

If a calculus becomes impacted in the opening of the ureter, the symptoms assume a more active character, the pains in the loins become much more severe, and the urine is then generally more or less charged with albumen or globules of blood.

In simple nephritis, the urine is either very slighly acid, or it is neutral, or even alkaline; and its sediment is usually composed of the urates or of phosphate of lime in an amorphous condition: in gouty nephritis, on the other hand, the urine is invariably more acid than in health, and the sediment is generally more or less decidedly crystallised. The symptoms in the latter case not unfrequently subside and disappear after the expulsion of a quantity of gravel or of a small calculus. The detraction of blood by leeches or cupping over the loins, or from the arm if there is much

---

* In the Foreign Periscope of the present number will be found a series of extracts from M. *Rayer's* work, on the anomalies, &c. in the structure of the kidneys,—*Rev*.

feverish irritation of the system, will however generally be necessary; and if the pain should continue after the gravelly matter has passed, it may be prudent to repeat the loss of blood; employing at the same time warm baths, poultices to the loins, and diaphoretic demulcent drinks.

When the renal region remains tender on pressure, after the feverish symptoms have entirely subsided, and when the urine is found at the same time to contain either purulent matter or a large quantity of mucus with or without an admixture of blood, we have reason to suspect the existence of one or more calculi in the pelvis or calices of the affected kidney. In such circumstances, the quantity of the purulent or mucous secretion may usually be much diminished by the use of balsamic and terebinthinate medicines. Alkalis and alkaline earths should be administered, as long as there is an excess of uric acid in the urine: their action seems to consist in combining with the acid of the urine, and thus forming salts which are much more soluble in the fluid than the acid itself. M. *Rayer* seems to have but little confidence in the decoction of the *pareira brava*, as an agent to restore a healthy state of the secretion. It has been, he says, from neglecting to test the urine and its deposits, that medical men have been so apt to attribute remedial properties to this and other substances for the relief of urinary complaints occurring in a gouty constitution.

Sir *B. Brodie* has drawn the attention of medical men to a set of cases, the symptoms of which very closely resemble those of nephritic colic, although differing from the latter in several respects. The disease, says this distinguished surgeon, usually attacks persons living a luxurious life, and prone therefore to the development of the gouty diathesis. The patient complains at first of a pain in the region of the kidneys, which afterwards extends towards the groin in the direction of the spermatic cord; at a later period, without any diminution of these symptoms, he finds that he has frequent calls to pass urine, and the effort to do this is often attended with sharp pains. The urine is usually scanty, of a deep colour, and more or less strongly acid, as indicated by its action on litmus paper. The symptoms will last for several days or even weeks, if no appropriate remedies are employed; but often they may be dissipated within a few hours by cupping over the loins, and by the use of frequently-repeated doses of colchicum. To prevent the return of such attacks, the most important precaution is to abstain from all wine and malt liquors,* and to use soda or Seltzer water, alone, or with the addition of a tea-spoonful of good brandy.

### *Rheumatic Nephritis.*

This variety of the disease has hitherto scarcely been noticed by any writer. Even in those cases where patients have died from organic affections of the heart and other internal viscera consequent upon rheumatism, no mention has almost ever been made of the state of the kidneys.

M. *Rayer* is however quite satisfied that these organs are liable to serious

---

* If the patient has not sufficient self-command to abstain from these beverages altogether, a glass or two of sound old sherry in water, or a tumblerful of the bitter East India ale—now so much in vogue—may be allowed. This ale, by the bye, is an admirable restorative during the convalescence from fevers, influenzas and such debilitating diseases.—*Rev.*

alterations during the progress of rheumatic affections. He gives the following description of his own observations:

"If the alteration was of recent date, the cortical substance of the kidneys was infiltrated in one or in several points with coagulable lymph; these solid depositions caused in almost every case a projection on the surface of the organ, where they looked as pale yellowish spots, often encircled with a ring of red. They varied much in size from that of a walnut to that of a hemp-seed; and some of them were usually found to penetrate for a considerable depth into the cortical substance. The lining membrane of the pelvis of the affected kidney occasionally exhibits beautiful arborisations of injected vessels; the size and weight of this viscus may also be greater than in health; and even minute depositions of purulent matter are sometimes observed.

When rheumatic nephritis has been of long standing, the kidney exhibits other appearances: the projections on its surface disappear, and are replaced with depressions, which are usually of a considerable size and of a yellow colour at the bottom. The investing membranes of the viscus adhere very firmly at these points, and can scarcely be detached from the cortical substance which they cover. Occasionally we meet with serous cysts in the cortical, and minute cartilaginous bodies in the tubular substance."

It is unnecessary to specify the symptoms of rheumatic nephritis, as there is nothing peculiar in this variety of the disease. It is only requisite that the medical man should be aware that there is a tendency to renal as well as to cardiac and other internal lesions in those who have suffered severely, or for a length of time, from arthritis. Occasionally the urine will be found to contain albumen; if pain in the back and testicle is present at the same time, the diagnosis is obvious that something is amiss with the kidneys.

The urine is usually very scanty and high-coloured in acute rheumatism; it contains a quantity of the urates in an amorphous state, and sometimes also a few crystals of uric acid—but never so abundantly as in cases of genuine gouty nephritis. M. *Rayer* lays much stress on this difference between the characters of the urinary deposit as a discriminating character between these two forms of the disease: the frequency of dyspeptic distress in the one form and of cardiac distress in the other should also be taken into account. This circumstance—the greater abundance of crystallized deposits in gouty than in rheumatic diseases—accounts not only for the frequency of gravel and nephritic colic in the one and the rarity of them in the other case, but also for the difference in the lesions of the kidneys discoverable on dissection in the two cases. In rheumatic as in gouty patients, attacks of complete ischuria are of occasional occurrence, and require prompt and vigorous treatment by bleeding, cupping, the use of the warm bath, &c.

Before quitting the subject of gouty and rheumatic nephritis, we must caution our readers against attempting to discriminate too nicely between these two forms of renal inflammation. It is often extremely difficult, nay impossible, to draw the line of distinction between some of the multiform varieties of gouty and rheumatic ailments; and the reason of this is no doubt that the two morbid states of the system are often associated and blended together in the same individual. Gout is unquestionably connected with the generation of an excess of acid in the fluids in consequence of imperfect digestion and assimilation of the food; and genuine rheumatism, we have strong reason to believe, seems to be connected with the existence of an excess in the fibrinous constituent of the blood. Now the former disease is very often grafted upon the latter, when the constitution of the patient, perhaps originally robust and plethoric, has become enfeebled, and the energies of the stomach and bowels impaired. In addition to his rheumatic pains, he begins to lose his appetite, he is troubled with flatulence and acidity after his meals, the urine is observed to deposit a larger quantity of sediment, and is found to be more strongly acid than in health. Such is the manner in which gouty ailments are slowly developed in a rheumatic habit of body; and, when this complication is once fairly established, it is almost impossible to decide what symptoms during life, and what lesions after death are attributable to the one, and what to the other disease. Let it be remembered too that neuralgic suffering is often superadded to the aches and distress arising from gouty and rheumatic causes; and thus the diagnosis is rendered more perplexing than ever. It is in such cases that the skill of the scientific physician is conspicuous. By nicely analyzing the different elements, so to speak, of the disease, he knows how to apply the appropriate remedies, either by combining several together so as to neutralise the various morbific causes at the same time, or by successively attacking and subduing them, one after the other.

Alkalis and colchicum are his chief agents for the relief of the gouty symptoms; local bleeding, mercury and guaiacum for that of the rheumatic, and anodynes and epispastics for that of the neuralgic distress. Strict attention to diet, and the occasional use of warm baths will greatly enhance the value of these remedies in all cases; more especially in those where the gouty symptoms are the predominant ones. We have been led into these digressive remarks by observing that one or two of the cases related by M. *Rayer* as illustrations of gouty nephritis seem to us more strictly to belong to the rheumatic series: take for example the following one.

A woman, 29 years of age, had been long subject to palpitations of the heart; its impulse was very strong, and the beats were very irregular, although no abnormal sound could be perceived. There was pulmonary emphysema on both sides; the patient was frequently distressed with dyspnœa, and she was also dropsical. On several occasions she had suffered from attacks of gout in the hands, which were enlarged, stiff, and deformed. She made no complaint of any urinary annoyance. By bleeding from the arm once, and the use of digitalis, the dropsy was dissipated, and the patient was in other respects much relieved. While in La Charité hospital, she had a very violent attack of dyspnœa; the face was anxious and of a blueish hue; the pulsations of the heart were rapid and extremely irregular, but not accompanied with any morbid sound; the right side of the chest was unusually resonant on percussion, and over its lower half a loud mucous rale was heard. The whole of the left side was dull, when struck with the fingers, and no respiratory murmur was perceptible: probably from the effusion of water. There was also œdema of the feet, and a certain degree of ascites. The hands, deformed by the gout, were very painful. These symptoms resisted every remedy that was tried, and the patient quickly sank.

On dissection, the pericardium was found to contain four or five ounces of fluid, but did not exhibit any traces of false membranes; the heart, especially its left ventricle, was greatly hypertrophied; the valves were but little affected. The lower lobe of the left lung was considerably compressed by fluid in this side of the chest; the right lung was emphysematous. Both kidneys were seriously altered by chronic inflammation. Instead of being smooth and regular on their surface, they were much deformed, rounded, and exhibited so many asperities as to make them resemble large mural calculi. When divided with the scalpel, the renal substance was found hard and contracted, as we observe in some cases of chronic nephritis, and was sprinkled over with particles of uric acid.

(Does not this case present the characters of rheumatic rather than of gouty disease?—*Rev.*)

*Pyelitis, or Inflammation of the Pelvis and Calices of the Kidney.*

By far the most common cause of this disease—which is much more frequently met with in the chronic than in the acute form—is the presence of calculi or gravel in the excretory apparatus of the organ. We have no space at present, and even if we had we should deem it a task quite unnecessary, to trace the course of this disease during its various periods of development. It is not by over-minute descriptions, so much as by a few practical hints strongly urged and illustrated, that the physician will be impressed with the importance of watching the progress of urinary complaints with more attention than he has hitherto been in the habit of paying to them. The careful inspection and examination of the urine will often enable him to detect the existence of latent disease, when every other means of diagnosis are utterly inefficient.

After dwelling at considerable length on the physical appearances of the urine in the early stages of pyelitis—the most conspicuous of which are the admixture of blood, mucous or muco-purulent matter, and the deposition of certain of the saline ingredients—M. *Rayer* makes the following remarks on its condition in the later periods of the disease:—

" The urine is usually bloody or purulent every time that it is voided, unless the disease is limited to one only of the kidneys, and the secretion from the diseased one be partially or entirely interrupted. We observe, however, great variations both in the frequency of the calls to pass the urine, and in its physical and chemical characters. When purulent urine coming from the pelvis of a kidney in the state of inflammation is retained only partially in its cavity, it becomes mixed, in variable proportions, with the urine from the other kidney, which may be at the time perfectly healthy.

The urine therefore may, in the course of the same day, exhibit very different appearances at different times; so that the medical man, if he trusted altogether to the state of the secretion as observed at one or two periods of the twenty-four hours, might be greatly misled in his judgment of the case. I have frequently seen, in cases of calculous pyelitis, the urine voided at one hour of the day highly charged with pus or blood, and at another hour perfectly clear and healthy—a state of things which can be explained only by supposing that the secretion came alternately from the diseased and from the sound kidney.

In some cases the suspension for a time of the unhealthy urine is always accompanied with an aggravation of the renal distress and with a febrile irritation of the system—in consequence, probably, of the ureter of the affected kidney becoming from some cause obstructed, and the urine therefore accumulating in its pelvis. The symptoms usually subside, when the voided urine again exhibits a purulent admixture.

It is necessary to be aware of the circumstance now mentioned, in order to avoid an error of diagnosis into which we should be apt to fall if we trusted to a single examination of the urine in twenty-four hours."

Let it also be remembered that the urine, when at all purulent, will be found to be albuminous on the application of heat, or by the addition of nitric acid; the amount, however, of the coagulum so produced is by no means proportionate to the quantity of purulent matter in the fluid.

The diagnosis of pyelitis is sometimes far from being easy. It may be mistaken for lumbago, for some kinds of nephritis, for simple nephralgia, for caries of the lumbar vertebræ, for psoitis, for obscure aneurism of the aorta, for some diseases of the ovaria, for disease of the colon, &c.—more especially as the urinary organs may be sympathetically affected at the same time.

It has been alleged by some writers that pyelitis may be distinguished from cystitis by the mere examination of the urine, when the other symptoms are obscure and ill-defined; that in the former disease the urine exhibits a genuinely purulent deposit, while in the latter it is usually only glairous and viscid. This remark is partially, but by no means universally, correct; for the purulent secretion of the kidneys may acquire a viscid character by a certain degree of alkalinity of the urine; and, on the other hand, that from the bladder, has not always this appearance.

When chronic pyelitis has existed for a length of time, and the excretion of the urine along the ureter is considerably obstructed either by the presence of a calculus in it or in the pelvis of the kidney, or from any other cause, a swelling may sometimes be distinctly felt in the lumbar region; and cases have been known where the process of suppuration and ulceration has been set up by Nature, until at length an abscess has been formed there, and, on the opening of this, one or more calculi have escaped from the wound in the loins. Acting upon the results of such cases, several surgeons have recommended that a deep incision should be made, when the swelling is distinct, until the distended pelvis of the kidney be opened, and thus an exit be provided for the discharge of the pent-up purulent matter and also of any foreign substance.

M. *Rayer* quotes several instances in which such an operation has been quite successful, and in which one or more calculi have been thus extracted; and it would seem that in one case, which occurred in his own practice, M. *Velpeau* performed it, with a satisfactory result, on a patient whose death appeared to be imminent.

As a matter of course, the greatest judgment must be exercised beforehand, to arrive at an accurate diagnosis; as the mere circumstance of the presence of a swelling over one of the kidneys, accompanied with symptoms of urinary distress is far from being a sufficient warrant for undertaking any operation: but, on the other hand, the surgeon is not to be deterred from resorting to it, when the ensemble of the symptoms points out that Nature is making an effort to relieve herself by establishing an abscess in the loins.

It is more than probable that in most of the successful cases, which have been recorded, the calculi had already escaped from the pelvis of the kidney through an ulcerated opening and were lodged in the cellular tissue exterior to it. In a curious case, related by *Roonhuysen*, a calculus was extracted from an extra-renal abscess or from the right kidney itself, and the patient continued to enjoy good health for a couple of years.

At this period, an inflammatory swelling appeared in the same place, and, on opening it with the knife, a second calculus was extracted. The wound healed, and the patient had no further trouble.

Some surgeons may prefer the use of caustic to that of the knife in opening such abscesses; and, judging from the successful application of this practice in the treatment of hepatic abscesses and cysts, it perhaps deserves the preference. Still no universal rule can be established; and much will depend on the peculiarities of each case.

*Renal Hæmorrhage.*

The admixture of blood with the urine is observed occasionally during the course of a variety of diseases, not only of the kidneys themselves, but also of the bladder and of the urethra. Several of the forms of nephritis are accompanied with sanguinolent urine: it is rather a common symptom in the early stage of albuminous nephritis, more especially where this disease has followed upon an attack of scarlatina. Even in its chronic stage, it is not uncommon to find, with the aid of the microscope, the presence of blood globules in the sediment of the urine; and, in the paroxysms or exacerbations of the malady, the proportion of these globules is often so great as to give the urine, which previously had been quite pale, a red or brownish hue.

In acute rheumatic and gouty nephritis also the urine is occasionally sanguinolent.

The formation of petechiæ and of depositions of blood in the substance and in the pelves of the kidneys, with genuine renal hæmaturia, are the usual phenomena of nephritis induced by morbid poisons.

Renal hæmorrhage is a not unfrequent symptom of *pyelitis*, especially of the calculous form of the disease; and also of cancerous and other morbid degenerations of the kidneys.

The secretion of blood from the urinary organs is observed in the course of certain constitutional diseases, and appears to depend upon an abnormal condition of the blood itself and probably of the bloodvessels also, without any distinct local malady of these organs. Thus in purpura and in scurvy the urine is often more or less decidedly sanguinolent. M. *Rayer* alludes to an interesting case which occurred in his own practice: it was that of a young man in whom there was hæmorrhage from four different mucous surfaces at the same time; viz. from the air-tubes, from the nostrils, from the intestines, and also from the urinary passages. *Latour* relates a similar case, which occurred in a young girl, who for four years frequently voided blood in all these ways, and ultimately recovered her health.

In hæmorrhagic diseases, the hæmaturia is often observed to cease for several days at a time, and again to make its appearance soon afterwards without any appreciable cause.

There is a form of renal hæmorrhage, rare indeed in any part of Europe,* but which seems to be of frequent occurrence in certain tropical countries; more especially in the Isles of France and Bourbon, and in Brazil.

M. *Chapotin*, in his topography of the Isle of France, thus expresses himself:

"In this island, the children of both sexes, from their most tender age, are subject to hæmaturia. In some, the discharge is continued and slight: in others it returns at intervals, and in varying quantities. It is usually unattended with pain or any symptom of constitutional disturbance; and the health of the infant remains good. It would be dangerous to check the discharge: and all that is necessary is to use means to strengthen the constitution, such as the use of cold baths and a nourishing diet.

This form of hæmaturia usually ceases about the period of puberty: but often it continues to a later period of life. Frequently it is replaced by attacks of nephritic colic, which seem to depend sometimes on the congestion of the bloodvessels, at other times on an excessive secretion of mucus, or on the presence of renal calculi.

---

* Although *endemic* hæmaturia has not been observed in man in Europe, it would seem that it occasionally occurs in some of the lower animals during certain seasons. It is necessary, however, to distinguish this disease from that which may depend upon the use of certain articles of food. Thus we are told by *Frank* that eating the *cystus laurifolius* is apt to induce hæmaturia in sheep, and that several species of *ranunculi* produce the same effects in cattle. M. *Drouard* says that in cows bloody urine is a not unfrequent occurrence in certain seasons during Spring.—(De l'hematurie dans l'espece bovine. Rec. de Med. Veterin. 1837.)

In corpulent persons I have often noticed a tendency to great congestion of the blood-vessels of the kidneys, indicated either by hæmaturia or by a suppression of the urine: the disease is sometimes owing to the cessation of a hæmorrhoidal discharge."

M. *Salesse*, himself a native of the Isle of France, and now a resident practitioner there, gave the following account of this endemic hæmaturia in 1834:

"Three fourths of the children in this island are affected with it. Masturbation, the use of spiced meats, &c. are its exciting causes; the bad quality of the water also has been blamed.......

When the blood comes from the kidneys, the patient is usually subject to attacks of nephritic colic: this in some cases is owing to the presence of renal calculi. The urine is mixed with blood; its colour is the same from the first jet to the last drops; and in some cases coagula come away with it. The only pain which is experienced is felt at the extremity of the glans. After any excess, whether in the pleasures of venery or of the table, the colour of the urine is usually more deep; the same effect is often induced by fatigue from long walking, dancing, and so forth....... When the blood comes from the bladder, the patient experiences pain in this region and about the anus; the perineum is the seat of an uneasy tension and sense of weight: and any excess will sometimes have the effect of rendering the urine less sanguinolent. The calls to pass urine are frequent; and coagula are not unfrequently mixed with it: the semen also is sometimes sanguinolent.

Persons affected with this hæmaturia are in general of a pale complexion and feeble constition."

Dr. *Salesse* quotes some very curious cases, most of which are reproduced in M. *Rayer's* work. The first is that of a gentleman, who had been subject to continual hæmaturia since he was seven years of age. In his 21st year, he made a voyage to France, in the hope that he might get rid of this complaint, although he did not experience any distress from it, except occasionally a sense of weight and tension in the perineum. While in Paris, the hæmaturia increased, and he consulted M. *Andral*, who recommended him to take a decoction of rhatany root. He had several attacks of ague, during which the urine was usually much freer from blood than it was at other times: the sulphate of quinine was freely administered. He always observed that the sanguineous admixture was less after the use of the cold bath. He never suffered from any pain or uneasiness in the renal region.

In another case, the patient, who had been affected with hæmaturia from his infancy, made a voyage from the Isle of France to France, to prosecute his studies. Soon after his arrival in Paris, his complaint disappeared, and did not recur during the whole time—a period of from six to seven years—that he resided there. He then went to the Mauritius; and a few months afterwards the hæmaturia returned, and still continues.

In some cases of this endemic hæmaturia, the urine is found to contain uric sand or gravel at the same time. M. *Rayer* observes that the reports of the preceding cases are not sufficiently minute, as to the nature of the urinary sediment, to enable us to determine whether there was not an admixture of the urates in them also. He very properly suggests that a chemical and microscopic examination of the urine should never be neglected.

A curious circumstance connected with this endemic disease is that the sanguineous state of the urine is occasionally followed by a milky, or as it has been called a chylous, state of the secretion.*

---

* Several illustrative cases are given at considerable length by M. *Rayer* from the works of M. *Chapotin* and others; but we cannot do more than refer to them at present.

This peculiar anomaly of the urinary secretion is not unfrequently met with in the Brazils, as well as in the Isles of France and Bonrbon. A very interesting case, which occurred in a gentleman a native of the former country, is related at great length in the Journal La Presse by M. *Caffe.** Some years ago an elaborate discussion on the nature of this disease, which is known under the name of milky diabetes, took place in the Medical Society of Rio-Janeiro. A committee was appointed for the purpose of investigating it with attention. It appears from their report that it is often met with in that city, more especially in hospital practice. It is more common in women than in men. Its duration is very variable; lasting sometimes for months and years, and then ceasing without appreciable cause. From the irregularity of its character, and the inconstancy of the morbid appearances found on dissection, Dr. *Simoni* (one of the physicians of the Misericorde Hospital) regards it as a nervous affection of the kidneys. He recommends the use of steel, valerian, &c. in the treatment of this disorder; but acknowledges that in some cases no remedy seems to be of any avail, and that the secretion will often assume a healthy appearance when nothing is done. The health of the patients is often surprisingly little affected during its continuance. The term *diabetes* is, Dr. *Simoni* remarks, improperly applied to this disease; as, in the majority of cases, there is no decided increase in the quantity of the urinary secretion. It seems to be generally admitted that a strengthening diet, tonic remedies and the use of the cold bath are the most useful means that can be employed.

M. *Rayer* remarks that he has several times had occasion to observe that hæmaturia, occurring in persons born and residing in Europe, has been followed by an albuminous state of the urine; but that he has never met with an instance of the chylous or albumino-fatty transformation except in persons born in tropical countries. The medical man must be on his guard not to mistake the whitish urine produced by the admixture of purulent matter with it, for this condition of the secretion. Purulent urine, if examined with the microscope, will be found to exhibit globules of pus diffused through it; and, when allowed to rest, a puriform sediment will be observed, while the urine above becomes less opaque and troubled. Chylous urine, on the other hand, is observed to contain either globules like those of the blood, or no globules at all (the albumino-fatty urine); and, after some hours' repose, the opaque matter rises to the surface and the urine below becomes somewhat less opaque.

Before quitting this subject, we may mention that hæmaturia seems to be of frequent occurrence in some other tropical countries, besides the Isle of France and the Brazils. We read that many of the soldiers of the French army in Egypt suffered from it. M. *Renoult* says that it was more common in the cavalry than in the infantry soldiers, and that even the horses were not exempted from it. He attributes it to the great diminution of the renal secretion, induced by the excessive perspirations, and to the irritation caused by the scanty acrid urine on the inner surface of the *bladder*. Severe exercise on horseback is occasionally apt to produce hæmaturia in some persons, even in temperate climates: M. *Aran* published a memoir on this subject in 1811.

We must here draw our remarks to a close. The deservedly great attention that has of late years been paid to the diseases of the urinary organs has rendered it quite necessary that every well-informed medical man should be acquainted with the recent standard publications on the subject. This has been our motive for giving a second review of M. *Rayer's* large and elaborate work—a work which reflects the highest credit on the author, and impresses on the reader a most favourable opinion of the present state of French medical literature.

---

* The report is inserted in the Number of the Medico-Chirurgical Review for April 1839, and will repay the trouble of a re-perusal.

